# Protein adsorption on spark plasma sintered 2d‐, 3d‐ and lamellar type mesoporous silicate compacts

**DOI:** 10.1049/iet-nbt.2020.0026

**Published:** 2020-09-22

**Authors:** Yoshiyuki Yokogawa, Keita Sasada, Koji Hirabayashi, Suguru Inamura, Takeshi Suyama

**Affiliations:** ^1^ Graduate School of Engineering Osaka City University 3‐3‐138 Sugimoto, Sumiyoshi‐ku Osaka Japan; ^2^ Research Division of Applied Material Chemistry Osaka Research Institute of Industrial Science and Technology 7‐1 Ayumino‐2 Izumi‐City 594‐1157 Japan

**Keywords:** silicon compounds, polymer blends, mesoporous materials, adsorption, plasma materials processing, proteins, sintering, powders, desorption, compaction, molecular biophysics, lamellar‐type MPS powder, PEO5PPO68PEO5, MPS‐la‐500, protein adsorption, lamellar type MPS compact, 3d‐cubic type MPS compact, spark plasma sintering, 2d‐hexagonal type MPS compacts, lamellar type mesoporous silicate compacts, lamellar type mesoporous silica compact, desorption, triblock copolymer, uniaxial pressure, PBS solution, protein solution, soaking time, temperature 500.0 degC, time 5.0 min, time 48.0 hour, time 4.0 hour, SiO_2_

## Abstract

The preparation of lamellar type mesoporous silica (MPS) compact through the spark plasma sintering (SPS) and the adsorption/desorption of protein onto MPS compact are reported to be compared with those onto 2d‐hexagonal and 3d‐cubic type MPS compacts. A lamellar‐type MPS powder (MPS‐la) was prepared using triblock copolymer, PEO_5_ PPO_68_ PEO_5_, and was compacted in a carbon die and heated at 500°C for 5 min under uniaxial pressure. The products are referred to as MPS‐la‐500. The MPS compacts keep the lamellar type mesoporous configuration. The adsorbed amount of protein onto MPS‐la‐500 was 100 mg/g, while that on MPS‐la was 130 mg/g, and the former decreased by 23%. However, its decreasing ratio of the protein adsorption on MPS‐la‐500 was less than those of 2d‐hexagonal and 3d‐cubic type MPS compacts, which were 73 and 34%, respectively. The released amount of protein into PBS solution from MPS‐la‐500, which was soaked in the protein solution for 48 h, increased with the soaking time, while those from 2d‐ and 3d‐type MPS compacts reached to plateau for 4 h of soaking. The lamellar type MPS compact was found to be easier to absorb and release proteins, which may be due to the large aperture of the mesoporous configuration.

## 1 Introduction

Attentions have been paid on nanostructured ceramic materials world‐wide due to their potential practical applications. Mesoporous silicate (MPS) materials have been subjected to a variety of applications, such as molecular sieves, catalysts, absorbent, carrier of bio‐molecules [1] and drugs [2], and so on [3, 4]. The periodically ordered pore structures of MPS are tailored to their optimum performances through self‐assembly of the structure‐directing agents (SDAs), cationic or non‐cationic surfactants. The typical cationic surfactant is CTAB ((C_16_ H_33_)N(CH_3_)_3_ Br, Cetyltrimethylammonium bromide) [5, 6] as a template of mesoporous silica MCM41. The typical non‐cationic surfactants are the triblock copolymers such as polyethy1ene oxide (PEO)–polypropy1ene oxide (PPO)–PEO (PEO–PPO–PEO), where the outer hydrophilic block is PEO and the inner hydrophobic block is PPO [7, 8]. The self‐assembly of the triblock copolymers, P‐123 [9], F127 [10, 11] and L121 [12] yields the 2d‐hexagonal, 3d‐cubic and lamellar type structure, depending on the molecular ratio of outer hydrophilic and inner hydrophobic blocks of the triblock copolymer.

The uniform pore diameters of 1.5–30 nm of MPS are close to the diameters of the target molecules, enzyme, antibody, gene expression protein and so on, and allow control of molecular adsorption based on size, surface potential and pH value of the solution and so on [1]. Proteins are easily denatured by a change of the surrounding conditions and should be immobilised in a certain carrier to be stabilised. The enclosure of the protein in such a well‐defined space of MPS materials may help to prevent denaturation of the protein [13].

For the environmental purification system, a variety of microorganisms are widely used due to their high‐cost performance, however, it requires space for the cultivation of microorganisms. Microorganism has their digestive enzymes to decompose environmental pollutants. The size of the enzyme is much smaller than that of microorganisms; the environmental purification system using enzymes can be scaled down and should be compact. Enzymes can be immobilised or encapsulated in/on polymeric or inorganic materials. The latter generally has chemical resistance, and activated carbon and silica gel are widely used, while MPS has some advantages of controlling pore size and shape, which should be suitable for enzyme adsorption.

Waste oil in sewage from homes and offices are generally treated using the grease trap, with the troublesome regular cleaning of the system. Also, oil‐degrading bacteria or their enzymes have been applied to the sewage, which is costly. The oil‐degrading enzyme, such as lipase, encapsulated MPS can be used repeatedly; as a result, it is less costly and has little effort.

The synthesis of MPS powders [14], films [15, 16] and fibres [17] has been intensively reported; however, the bulk MPS materials are rarely reported, because it may not be possible for the conventional processing (sintering) at high temperature to prevent cleavage and collapse of the mesoporous configurations of MPS materials. The bulk MPS materials may be applicable for the parts of pressure resistance, such as filler in the water treatment system.

Recent, partial fusion of MPS powders was reported by pulsed current processing (PCP) under a uniaxial pressure of 20 MPa [18]. The PCP technique is now commonly called spark plasma sintering (SPS), allows the rapid production of mechanically stable monoliths. MPS compact was also prepared using a unique process of SPS, a sintering technique with simultaneous application of heat, uniaxial pressure, and pulsed current in a carbon die, which involving a combination of rapid heating and applied pressure and is capable to ceramic powders into the mechanically stable dense body at relatively low temperature. When heating MPS compacts, first, partial fusion of MPS particle may occur as an initial sintering process, and then densification may proceed by heating at 600°C and over. Previous reports revealed that the mesoporous configuration was partially broken at 700°C [19], broken at 900°C and disappeared at 1020°C to produce transparent silica glass materials [20] by SPS processing. As mentioned above, the previous studies aimed at the densification of silicate compacts made from highly porous powders like MPS materials by heating at 600°C and over.

The authors tried to synthesise a 2d‐hexagonal type MPS compact using an SPS technique without any affecting mesoporous configuration, and successfully obtained 2d‐hexagonal MPS compact by heating at 500°C for 5 min under a uniaxial pressure of 25 MPa [19].

In this paper, 2d‐hexagonal, 3d‐cubic and lamellar type of MPS powders were prepared by sol–gel method with block copolymers and MPS compacts were formed by SPS processing at 500°C using three types of MPS powders, such as lamellar, 2d‐hexagonal and 3d‐cubic type MPS powders, to keep the mesoporous configurations, and the comparison of adsorption/desorption of lipase, an enzyme that catalyses the hydrolysis of fats, onto the three types MPS powders and MPS compacts was investigated. The lipase encapsulated MPS compacts are expected for the use of water treatment of waste oil in sewage from homes and offices.

## 2 Materials and method

### 2.1 Materials

MPS powders were prepared using triblock copolymer, PEO_a_ PPO_b_ PEO_c_ (P‐123: *a*  = 20, *b*  = 70, *c*  = 20, L‐121: *a*  = 5, *b*  = 68, *c*  = 5 and F‐127: *a*  = 98, *b*  = 67, *c*  = 98) as SDA. TEOS was added into the 2 M HCl solution containing SDA and further stirred for 1 h at 35°C. The solution was hydrothermally treated at 100°C for 24 h and the product was filtered, dried at 80°C for 24 h and calcined at 550°C to form MPS powder. The MPS powders prepared using P‐123, L‐121 and F‐127 are referred to as MPS‐2d, MPS‐la and MPS‐3d, respectively.

The SPS equipment used in this study was Dr.Sinter‐SPS1020, Sumitomo Materials Co. MPS powder was compacted in a carbon die and heated at a rate of 100°C/min up to 500°C for 5 min under uniaxial pressure of 25 MPa. The MPS compacts prepared at 500°C using MPS‐2d are referred to as MPS‐2d‐500, and MPS compacts using MPS‐la and MPS‐3d are named similarly.

### 2.2 Characterisation

The crystal phases of the MPS products were examined by an X‐ray powder diffraction method using an X‐ray diffractometer (RINT 2000, Rigaku Co., Japan) at 40 KV and 20 mA with CoKα radiation. Identification of the phases was achieved by comparing the diffraction patterns with ICDD (JCPDS) standards.

The MPS powders or compacts were dried in a vacuum at 150°C for 24 h to remove the adsorbed water and Brunauer–Emmett–Teller (BET) surface areas and Barett–Joyner–Halenda (BJH) pore size distributions of the products were measured by AUTOSORB‐1 (Yuasa Ionics Inc., Japan).

SEM and observation of the MPS powders or compacts were performed using FE‐SEM (JSM‐6500FS, Jeol Ltd., Japan) operated at 15 kV. To avoid the charging problem during observation, Os was deposited onto the sample surface using Os plasma coater (OPC‐60A, Filgen Inc., Japan).

Commercial lipase (F‐AP, Amano Enzyme Inc., Japan, isoelectric point: 4.0, 33 kDa) was put into phosphate buffer saline (PBS, pH 7.0) solution to prepare 2.0 g/l lipase solution, to which 30 mg of the MPS powders or compacts were put, and cooled at 4°C for 48 h with stirring. Then, the solution was filtered to remove the MPS powders or compacts and the adsorbed amount of lipase on MPS powders or compacts was determined by absorbance of the lipase solution at wavelength 279 nm using UV‐spectrometry (V‐500, JASCO Co., Japan). The MPS powders or compacts soaked in the lipase solution for 48 h, taken from the lipase solution, washed, and then put into the pure PBS solution. The concentrations of lipase in the PBS solution were measured to obtain the amount of released lipase from MPS powders or compacts in order to investigate the amount of lipase remaining in MPS powders or compacts.

## 3 Results

### 3.1 MPS powders and MPS compacts prepared by SPS processing

**Fig. 1:**
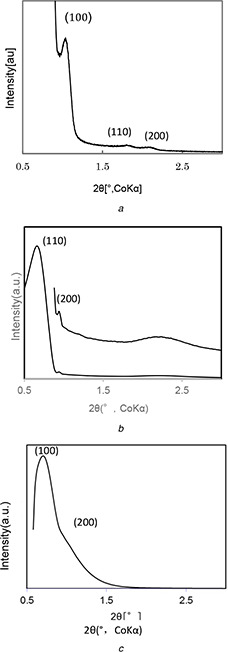
XRD profiles of MPS powders, ***(a)*** MPS‐2d, ***(b)*** MPS‐3d, ***(c)*** MPS‐la

Fig. 1 shows the XRD profiles of the 2d‐hexagonal, 3d‐cubic and lamellar type MPS powders (MPS‐2d, MPS‐3d, and MPS‐la) are characteristic of those of 2d‐hexagonal, 3d‐cubic and lamellar type MPS, respectively. A strong peak with d(100) spacing and two weak peaks with d(110) and d(200) spacings are observed in MPS‐2d, the peaks of d(110) and d(200) spacing in MPS‐3d, and the peaks of d(100) and d(200) in MPS‐la are also observed to confirm the formation of 2d‐hexagonal, 3d‐cubic and lamellar type of MPS structures, respectively.

**Fig. 2:**
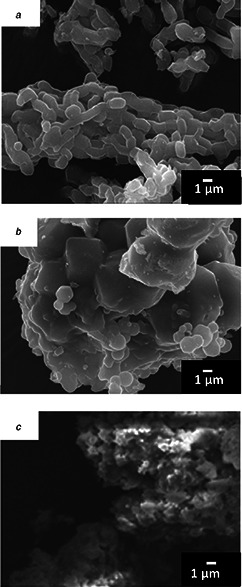
SEM images of MPS powders ***(a)*** MPS‐2d, ***(b)*** MPS‐3d, ***(c)*** MPS‐la

The aggregates composed of powders which exhibit characteristic morphologies in shape, as shown in Fig. 2. The MPS‐2d powders are aggregates composed of cylindrical particles around 100 nm long, which are connected in the long axis direction. The MPS‐3d are aggregates of cube‐like particles, and MPS‐la are those of flake‐like particles.

The individual particles can be observed in the spark plasma sintered MPS compacts, however, the interstices between MPS powders in MPS compacts are narrowed, and it was found that the MPS compacts prepared through SPS processing are a mass of partial fused MPS powders.

The pore volume of MPS compacts (MPS‐2d‐500, MPS‐3d‐500 and MPS‐la‐500) decreased by 21, 9 and 12% compared to those of MPS powders (MPS‐2d, MPS‐3d and MPS‐la), respectively. While the surface areas of MPS compacts (MPS‐2d‐500, MPS‐3d‐500 and MPS‐la‐500) decreased by 34, 3.7 and 16% compared to those of MPS powders (MPS‐2d, MPS‐3d and MPS‐la), respectively, as shown in Table 1.

**Table 1:** Pore diameter, surface area, pore volume of on MPS powders and MPS compacts and adsorbed amount of lipases

Sample name	Pore diameter, nm	BET surface area, m^2^ /g	Pore volume, cc/g	Adsorbed amount of lipase, mg/g
MPS‐2d	9.0	866	1.14	163
MPS‐2d‐500	9.0	670 (−34%)	0.90 (−21%)	45 (−73%)
MPS‐la	11.8	409	0.752	130
MPS‐la‐500	9.9	394 (−3.7%)	0.664 (−12%)	100 (−23%)
MPS‐3	6.3	838	0.651	42
MPS‐3d‐500	5.8	705 (−16%)	0.589 (−9%)	28 (−34%)

The digits shown in () are the decreasing rate (%) in the surface area or the pore volume of MPS powder/MPS compact.

Secondary particle sizes of MPS‐2d, MPS‐3d and MPS‐la are almost the same and around 10–20 µm; however, the size and shape of the primary particle should affect the densification of MPS compacts. The pore volume and surface area of MPS‐2d‐500 show a large decrease, which may be due to the small primary particle size of MPS‐2d. The primary particle of MPS‐3d is a large cube‐like particle, and that of MPS‐ls is a small flake particle in shape, and reduction rates of MPS compact are different, as shown in Table 1.

Fig. 2 shows the pore size distribution of 2d‐hexagonal, 3d‐cubic and lamellar type MPS powders and MPS compacts. The peak profiles of 2d‐hexagonal and 3d‐cubic type MPS powders and MPS compacts are sharp, while that of the lamellar type of seem to be broad. The mesopores of lamellar type MPS vary widely from 10 to 20 nm. The peak top intensities of MPS compacts are smaller than those of MPS powders, which may be due to the partial fusion of MPS powders during SPS processing.

**Fig. 3:**
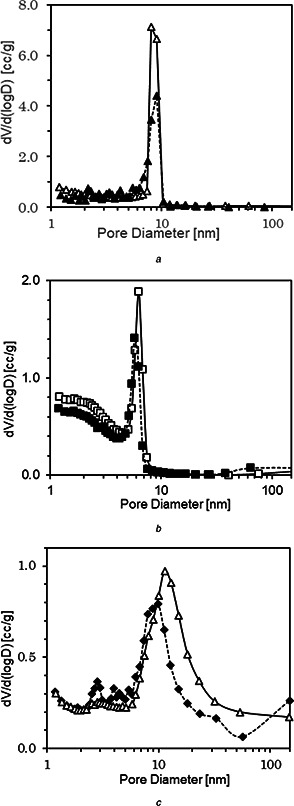
Pore size distribution of ***(a)*** MPS‐2d (white up‐pointing triangle) and MPS‐2d‐500 (black up‐pointing triangle), ***(b)*** MPS‐3d (white square) and MPS‐3d‐500 (black square), ***(c)*** MPS‐la (white circle) and MPS‐la‐500 (black circle)

Fig. 3 shows the pore size distribution of 2d‐hexagonal, 3d‐cubic and lamellar type MPS powders and MPS compacts. The peak profiles of 2d‐hexagonal and 3d‐cubic type MPS powders and MPS compacts are sharp, while that of lamellar type of seem to be broad. The mesopores of lamellar type MPS vary widely from 10 to 20 nm. The peak top intensities of MPS compacts are smaller than those of MPS powders, which may be due to the partial fusion of MPS powders during SPS processing.

**Fig. 4:**
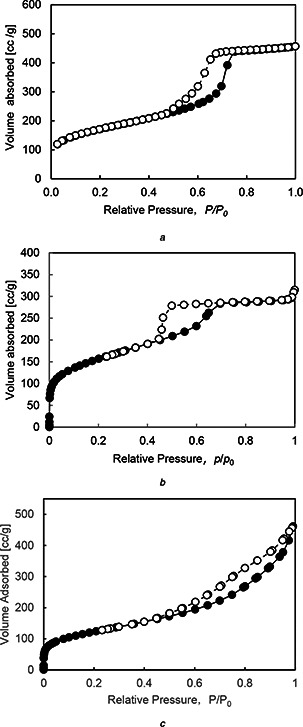
Adsorption isotherm (adsorption‐desorption curve) of ***(a)*** MPS‐2d‐500, ***(b)*** MPS‐3d‐500, ***(c)*** MPS‐la‐500

Fig. 4 shows the adsorption isotherm (adsorption–desorption curves) of 2d‐hexagonal, 3d‐cubic and lamellar type MPS compacts. The profiles of MPS‐2d‐500 and MPS‐3d‐500 can be classified as a cylinder‐shaped hole and that of MPS‐la‐500 as a wedge‐shaped hole, according to the IUPAC model, from which can be deduced the differences of the profiles of pore size distributions between MPS‐2d‐500, 3d‐500 and MPS‐la‐500.

### 3.2 Protein adsorption

**Fig. 5:**
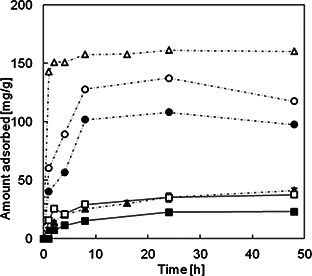
Time‐dependent adsorbed amount of lipase on MPS particles and MPS compacts. Black up‐pointing triangle：MPS‐2d‐500，black circle：MPS‐la‐500 and, black square：MPS‐3d‐500

Fig. 5 shows the time‐dependent adsorbed amount of lipase on MPS powders and MPS compacts. The amount of lipase adsorbed on the MPS powders and compacts increased with an increase of soaking time in the lipase solution.

The amount of lipase adsorbed on MPS powders (MPS‐2d, MPS‐3d and MPS‐la) for 48 h was 163, 42 and 130 mg/g, respectively. That on MPS‐3d was relatively lower compared to those on MPS‐2d and MPS‐la, which may be due to the small pore size of MPS‐3d with respect to the size of lipase.

The amount of lipase adsorbed on MPS compacts (MPS‐2d‐500, MPS‐3d‐500 and MPS‐la‐500) for 48 h decreased to 45, 28 and 100 mg/g, respectively. The decreasing ratio of MPS‐2d compact remarkably decreased by 73%, that on MPS‐3d compact by 34%, and that on MPS‐la compact by 23%.

Table 1 summarises the pore diameter, BET surface area, pore volume of MPS powders and MPS compacts, and adsorbed amount of lipase on MPS powders and MPS compacts.

The pore volumes of MPS compacts (MPS‐2d‐500, MPS‐3d‐500 and MPS‐la‐500) decreased by 21, 9 and 12% compared to those of MPS powders (MPS‐2d, MPS‐3d and MPS‐la), respectively. While the surface areas of MPS compacts (MPS‐2d‐500, MPS‐3d‐500 and MPS‐la‐500) decreased by 34, 3.7 and 16% compared to those of MPS powders (MPS‐2d, MPS‐3d and MPS‐la), respectively.

In the SPS processing, MPS powders encapsulated in a carbon die were heated under pressure, which may compress the outside of MPS compacts, leading to the densification and smaller openings of the outer side MPS compacts. Due to that, the decreasing rates of adsorbed amounts of lipase on MPS compacts are larger than those of pore volumes and surface areas.

The decreasing rate of the adsorbed amount of lipases on MPS‐la‐500 is smaller than those on MPS‐2d‐500 and MPS‐3d‐500, which may be related to low decreasing rates of surface area and pore volume.

**Fig. 6:**
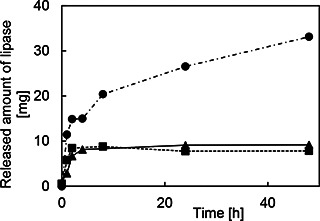
Time‐dependent released amount of lipase on MPS particles and MPS compacts. White up‐pointing triangle：MPS‐2d，black up‐pointing triangle：MPS‐2d‐500，white circle： MPS‐la, black circle：MPS‐la‐500, white square：MPS‐3d, black square： MPS‐3d‐500

Fig. 6 shows the time‐dependent released amount of lipases from MPS compacts as a function of soaking time in PBS solution. MPS compacts were immersed in a lipase solution for 48 h prior to soaking in PBS solution. The released amount of lipases from MPS‐2d‐500 and MPS‐3d‐500 reached a plateau for 2 h of soaking time, while that from MPS‐la‐500 is increasing with the soaking time. The lamellar type of MPS compacts seems to easily release the protein.

## 4 Discussion

**Fig. 7:**
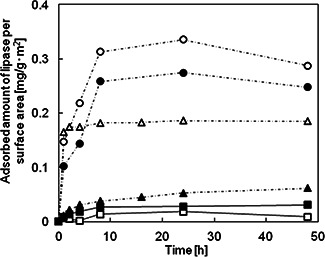
Time‐dependent adsorbed amount of lipase on MPS particles and MPS compacts per surface area. White up‐pointing triangle：MPS‐2d，black up‐pointing triangle：MPS‐2d‐500，white circle：MPS‐la, black circle：MPS‐la‐500, white square：MPS‐3d, black square： MPS‐3d‐500

The decreasing rates of the adsorbed amount of lipases on MPS compacts arrange in the following order such as 2d>>3d>lamellar, which does not the same as the order of the decreasing rates of pore volumes but the same order of those of surface areas, suggesting that the amount of adsorbed amount of lipase may be related with the number of accessible adsorption sites on the mesopores, which should be related to the surface areas of MPS compacts. As previously reported [21], the protein adsorption should proceed along with three steps; the first step as the adsorption on the surface of aggregates, the second step as the protein penetration in the inter‐particles of the aggregates and the third step as the diffusion into the mesopores of individual single particles. The number of accessible adsorption sites on the mesopores should depend on the structure of the sintered body composed of different types of MPS powders. The adsorbed amount of lipases per MPS surface areas along per MPS weight is shown in Fig. 7.

**Fig. 8:**
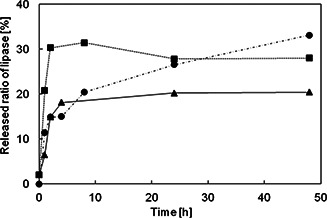
Time‐dependent released rates of lipase from MPS compacts, immersed in lipase solution for 48 h, as a function of soaking time in PBS solution. Black up‐pointing triangle：MPS‐2d‐500，black circle：MPS‐la‐500, black square：MPS‐3d‐500

Fig. 8 shows the time‐dependent released rates of lipase from MPS compacts as a function of the soaking time in the PBS solution. The released rates were calculated by dividing the released amount by the adsorbed amount of lipases for 48 h of soaking in the lipase solution. Those from 3d‐cubic type MPS compacts are higher and reached a plateau for 2 h of the soaking time. Those from the 2d‐hexagonal type of MPS compacts also reached a plateau for 2 h, but the released rate was lower, suggesting that the lipase was immobilised in the mesopores of the 2d‐hexagonal type. The SPS processing may promote MPS particle bindings and the pore volumes decreased by MPS particle bindings. It may produce the narrow interstices in the MPS compacts along with the shrinkage of MPS compact. As a result, less amount of lipase may reach the inner side of 2d‐hexagonal and 3d‐cubic type MPS compacts, while the released rate of lipase from lamellar type MPS compact is increasing with the soaking time.

The protein adsorption onto MPS materials proceeds through three steps; the adsorption on the external surface of MPS powders, diffusion between MPS powders, and diffusion inside mesopores to reach an equilibrium of protein adsorption [22, 23, 24]. Therefore, the lipase may exist in the vicinity of the surface of the MPS compact. While the lamellar type MPS compact was found to be easy to release lipase, which may be due to the large aperture of the lamellar configuration.

## 5 Conclusion

MPS bulk compact with mesoporous configuration was successfully prepared by the SPS technique. The adsorbed amount of lipase on spark plasma sintered MPS compact was reduced, and its reduction ratio was lower than that of the pore volume. The adsorbed amount of protein on MPS compacts does not depend on the pore diameter, BET surface area, pore volume, but on the interstices between MPS particles in MPS compacts. MPS compacts prepared by SPS show high capacity for protein and are expected to a protein carrier. MPS compacts with three types of mesoporous configurations were successfully prepared by SPS processing, and they are expected to be promising as a protein carrier for bi‐medical applications.
